# Supplementary material for: The role of graph topology in the performance of biomedical Knowledge Graph Completion models
Source: Bioinformatics. 2025 Oct 7;41(10):btaf547. doi: 10.1093/bioinformatics/btaf547 (PMC12560816; doi:10.1093/bioinformatics/btaf547)
Supplement: btaf547_Supplementary_Data [file btaf547_supplementary_data.pdf]

Supplementary Material for "The Role of Graph Topology in the Performance of Biomedical Knowledge Graph Completion Models"

Alberto Cattaneo[1], Stephen Bonner[2], Thomas Martynec[2], Edward Morrissey[2], Carlo Luschi[1], Ian P Barrett[2], and Daniel Justus[1]

[1] *Graphcore Research, Graphcore, Bristol, UK*
[2] *Data Sciences and Quantitative Biology, Discovery Sciences, R&D, AstraZeneca, Cambridge UK*

1 Dataset and Model Properties

Table S1: Properties of the considered datasets.

Graph	# Entities	# Relations	# Triples	Avg node degree
Hetionet	45,158	24	2,250,197	99.66
OpenBioLink	184,635	28	4,563,405	49.43
PharMeBINet	2,653,751	208	15,883,653	11.97
PharmKG	188,296	39	1,093,236	11.61
PrimeKG	129,375	30	4,050,064	62.61
FB15k-237	14,541	237	310,116	42.65

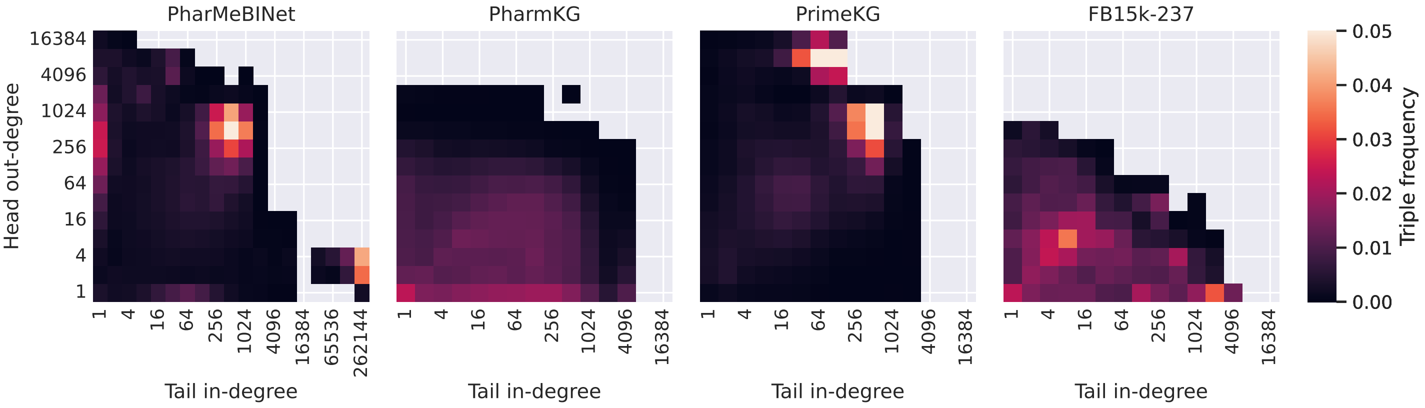

Figure S1: Relative frequency of triples when grouped by head out-degree and tail in-degree of the same relation type.

Table S2: Scoring functions and their ability to model four fundamental relation properties: S = Symmetry; INF = Inference; INV = Inversion; C = Composition. For RotatE we assume d even and denote by $\mathbb{C}^{\frac{d}{2}}$ the vector space $\mathbb{R}^d = (\mathbb{R} \oplus i\mathbb{R})^{\frac{d}{2}}$ with the structure of $\mathbb{R}$-algebra induced by the product of complex numbers. $p \in \{1, 2\}$; $\circ$ denotes the Hadamard product, $*w$ is the application of a convolutional filter w.

Model	Scoring function		S	INF	INV	C
TransE	$-\|\boldsymbol{h}+\boldsymbol{r}-\boldsymbol{t}\|_p$	$\boldsymbol{h}, \boldsymbol{r}, \boldsymbol{t} \in \mathbb{R}^d$	✗	✓	✓	✓
RotatE	$-\left\|\boldsymbol{h} \circ e^{i\boldsymbol{r}}-\boldsymbol{t}\right\|_p$	$\boldsymbol{h}, \boldsymbol{t} \in \mathbb{C}^{\frac{d}{2}}, \boldsymbol{r} \in \mathbb{R}^{\frac{d}{2}}$	✓	✓	✓	✓
DistMult	$\langle \boldsymbol{r}, \boldsymbol{h}, \boldsymbol{t} \rangle$	$\boldsymbol{h}, \boldsymbol{r}, \boldsymbol{t} \in \mathbb{R}^d$	✓	✓	✗	✗
TripleRE	$-\|\boldsymbol{h} \circ \boldsymbol{r_h} + \boldsymbol{r_m} - \boldsymbol{t} \circ \boldsymbol{r_t}\|$	$\boldsymbol{h}, \boldsymbol{r_h}, \boldsymbol{r_m}, \boldsymbol{t}, \boldsymbol{r_t} \in \mathbb{R}^d$	✓	✓	✓	✓
ConvE	$\langle \mathrm{MLP}([\boldsymbol{h}, \boldsymbol{r}] * w), \boldsymbol{t} \rangle$	$\boldsymbol{h}, \boldsymbol{r}, \boldsymbol{t} \in \mathbb{R}^d, w \in \mathbb{R}^{3\times 3}$	✓	✓	✓	✓

2 Details on Experimental Setup and Hyperparameter Selection

All datasets were randomly split into training, validation and test set (80% / 10% / 10%; in the case of PharMeBINet, 99.3% / 0.35% / 0.35% to mitigate the increased inference cost on the larger dataset). To ensure comparability across KGs, this random split was used even if pre-defined training, validation and test sets were provided with a dataset. We adopted log-sigmoid loss with negative adversarial sampling (Sun et al., 2019) and margin 12.0, and the Adam optimiser (Kingma and Ba, 2015) for updating parameters. During training we always used negative sample sharing (Cattaneo et al., 2022). All experiments were performed on Graphcore IPUs using the BESS framework[1] (Cattaneo et al., 2022). A fixed batch size of 768 triples per device (192 for PharMeBINet) was adopted, while the embedding size for entities and relations was chosen for each KG and each scoring function independently to maximise the memory utilisation of a Bow-2000 IPU machine with 4 IPU processors (in the case of PharMeBINet, a Bow Pod_{16} with 16 IPUs). This is to ensure a fair comparison between scoring functions with different memory costs. For some scoring functions, especially DistMult, the memory footprint is typically dominated by the model parameters, allowing a larger hidden size for smaller KGs. For other scoring functions, especially TripleRE and ConvE, memory is typically dominated by activations, resulting in a similar hidden size for differently sized KGs. The learning rate, the norm used by the scoring function (L1 or L2) and the number of negative samples were determined by a hyperparameter sweep, based on the validation MRR (Table S3). The full configurations can be found in the training scripts, released with the code[2].

Table S3: Experiment hyperparameters for different datasets. *Hetionet_same* refers to the alternative experimental configuration used in sec. *Case Study: Effect of Additional Training Data*.

Graph	Model	Hidden size	Learning Rate	Scoring Norm	# Negative samples / positive
Hetionet	DistMult	2048	0.0003	-	16
	RotatE	512	0.001	L2	16
	TransE	1024	0.0001	L1	16
	TripleRE	384	0.0001	L1	16
	ConvE	676	0.001	-	16
Hetionet_same	DistMult	300	0.0003	-	16
	RotatE	128	0.003	L2	16
	TransE	256	0.0003	L1	16
	TripleRE	256	0.0001	L1	16
OpenBioLink	DistMult	768	0.0003	-	16
	RotatE	256	0.003	L2	16
	TransE	512	0.0001	L1	16
	TripleRE	256	0.001	L2	16
	ConvE	529	0.001	-	16
PharMeBINet	DistMult	300	0.003	-	16
	RotatE	128	0.001	L2	16
	TransE	256	0.00003	L1	16
	TripleRE	256	0.0001	L2	16
	ConvE	256	0.0001	-	16
PharmKG	DistMult	768	0.003	-	16
	RotatE	384	0.003	L2	16
	TransE	768	0.0001	L1	16
	TripleRE	384	0.0003	L1	16
	ConvE	529	0.001	-	16
PrimeKG	DistMult	1024	0.0003	-	16
	RotatE	384	0.001	L2	16
	TransE	768	0.0001	L1	16
	TripleRE	256	0.0001	L1	16
	ConvE	576	0.0003	-	16
FB15k-237	DistMult	4096	0.001	-	16
	RotatE	1024	0.003	L2	16
	TransE	2048	0.0001	L1	16
	TripleRE	256	0.001	L1	16
	ConvE	676	0.001	-	16

[1] https://github.com/graphcore-research/bess-kge

[2] https://github.com/graphcore-research/kg-topology-toolbox/tree/main/the_role_of_graph_topology_paper/train

3 Additional Results

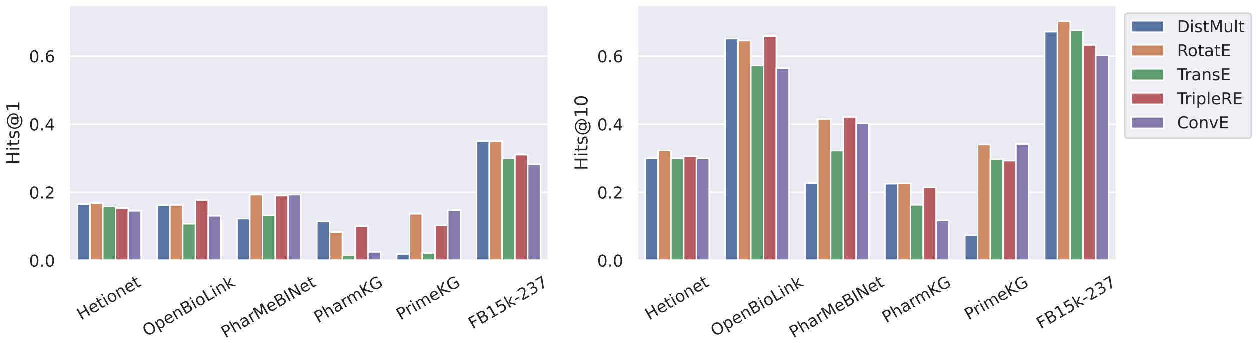

Figure S2: Hits@1 and Hits@10 on the test split achieved by the KGE models, for the six datasets.

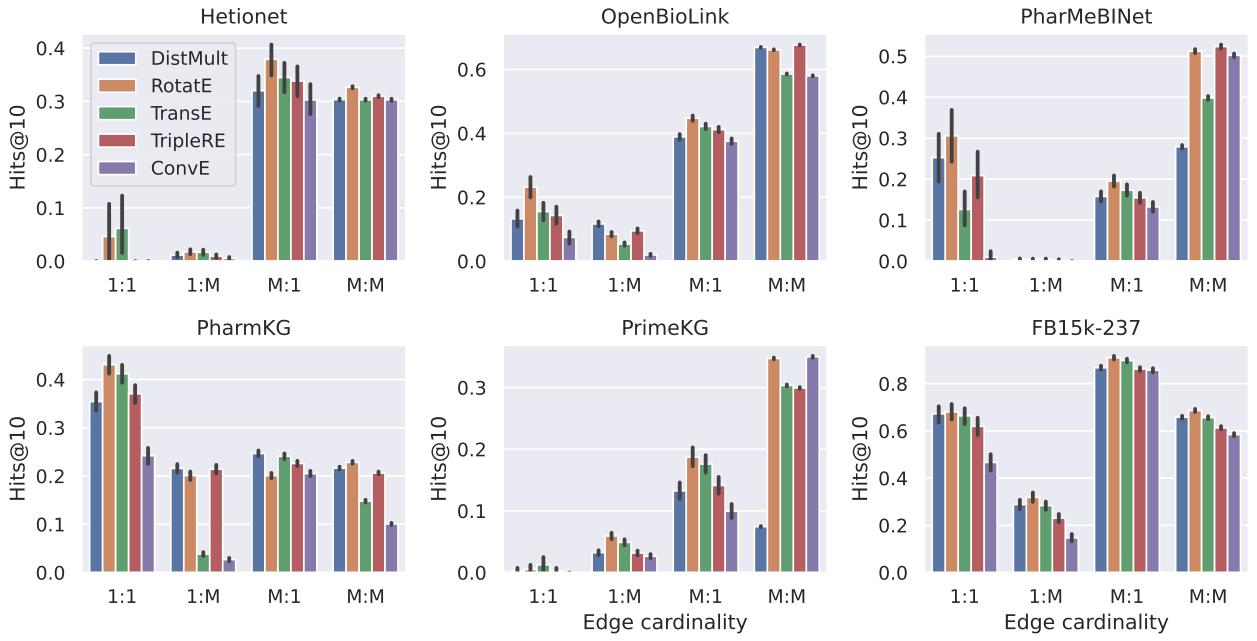

Figure S3: Effect of edge cardinality on Hits@10.

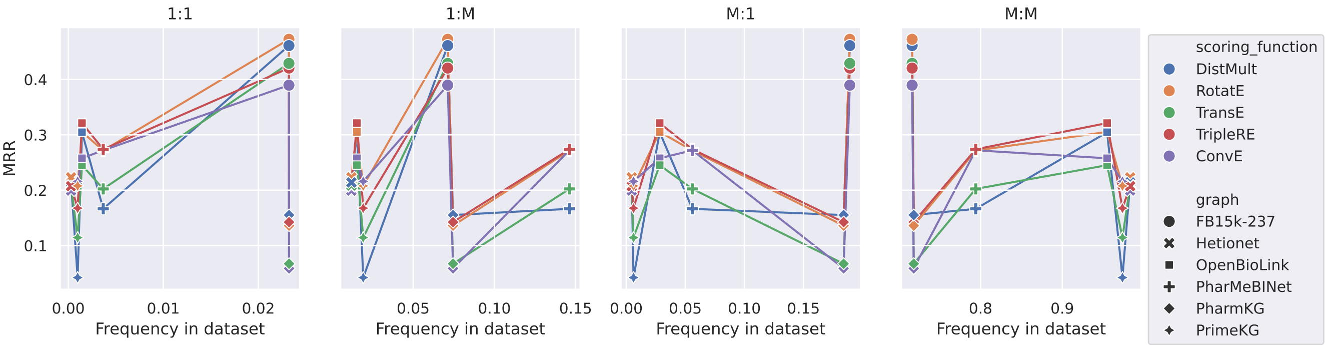

(a)

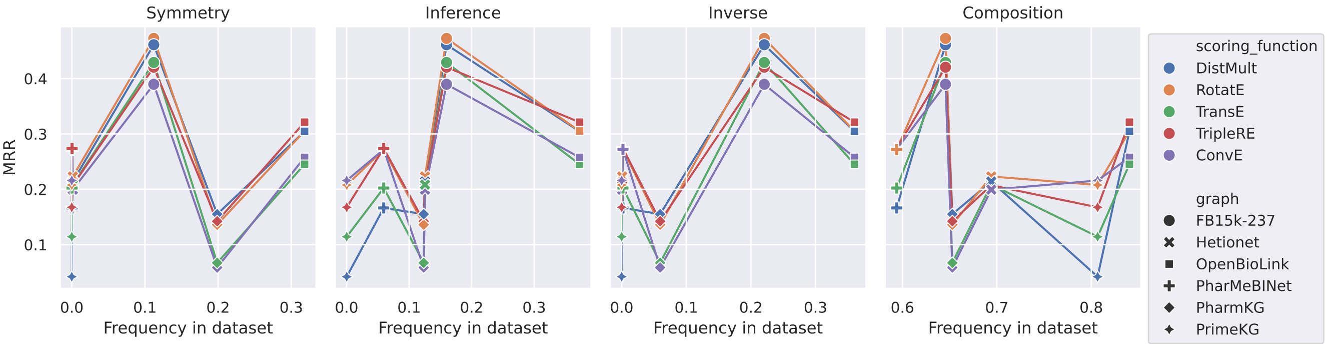

(b)

Figure S4: Mean reciprocal rank (on the test set) plotted as a function of (a) the frequency of edge cardinalities (i.e., the fraction of triples in a dataset of a given edge cardinality, see fig. 4) and (b) the frequency of edge topological patterns (see table 1).

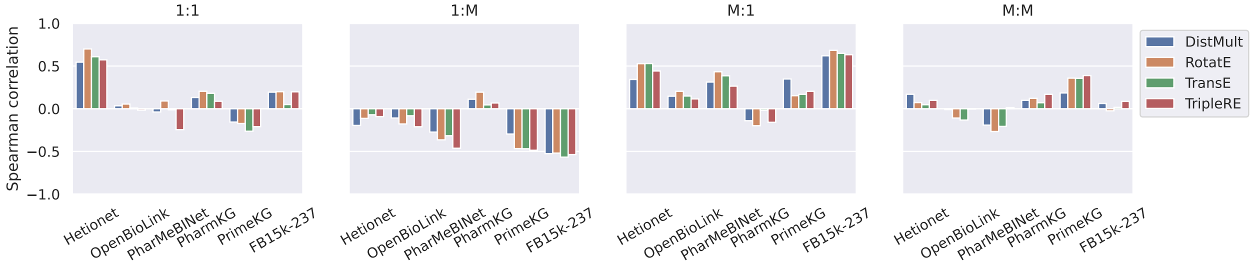

(a)

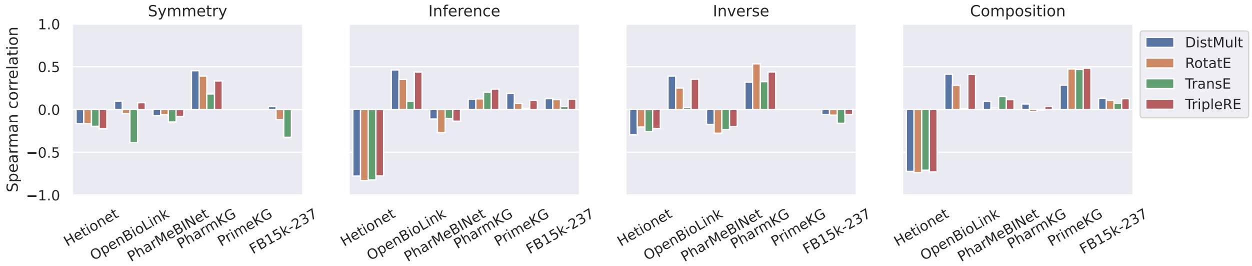

(b)

Figure S5: Spearman-rank correlation between the average MRR of a relation type and the average frequency of edge cardinalities (a) and of topological patterns (b) in that relation type.

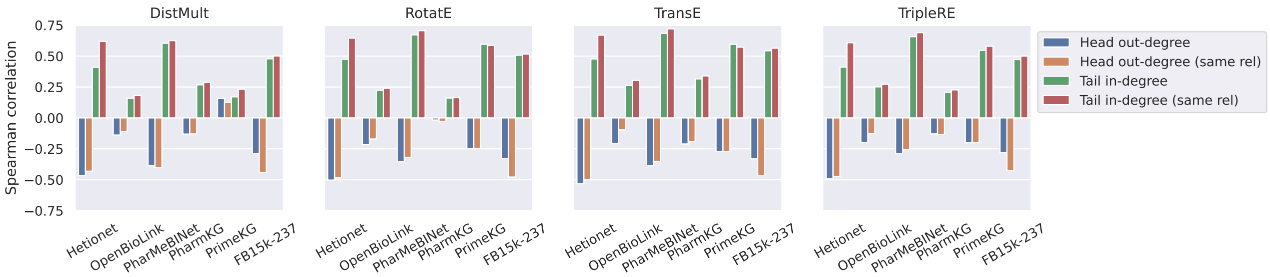

Figure S6: Spearman-rank correlation between MRR of individual triples and the out-degree of the head node as well as in-degree of the tail node.

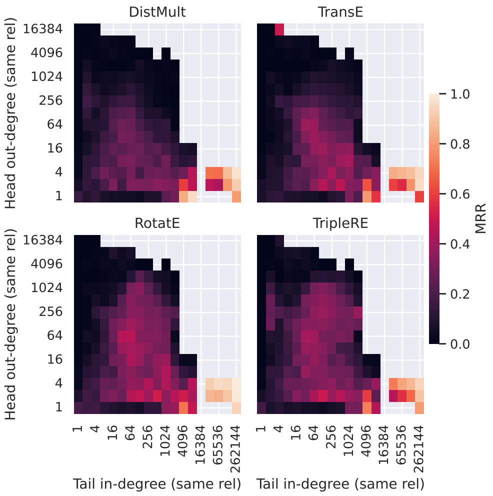

(a) PharMeBINet

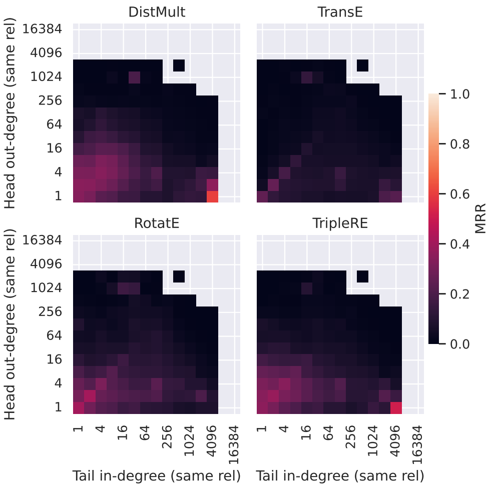

(b) PharmKG

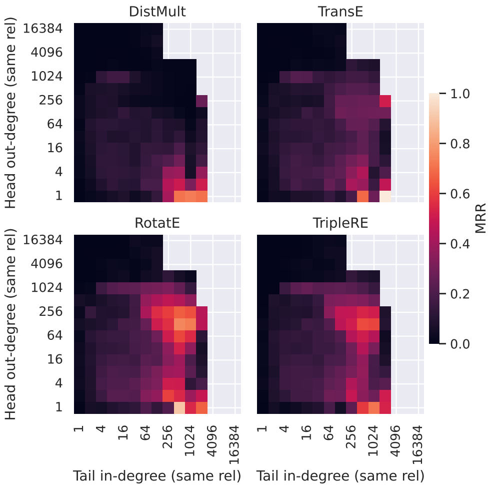

(c) PrimeKG

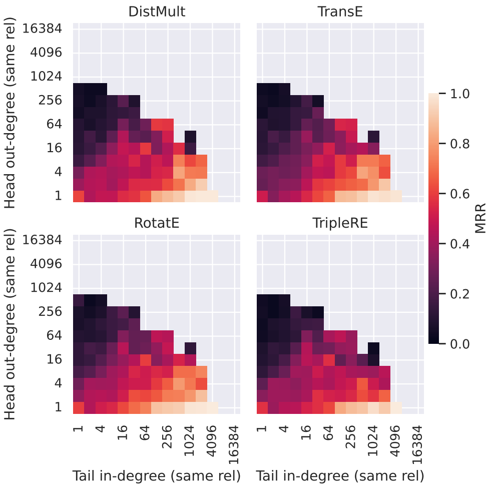

(d) FB15k-237

Figure S7: Effect of head out-degree and tail in-degree on MRR for additional datasets.

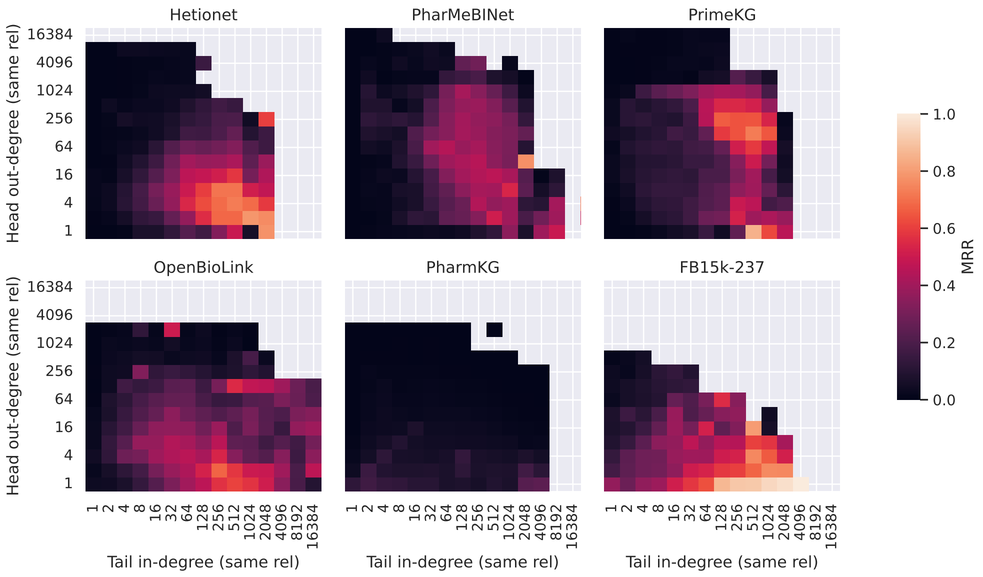

Figure S8: Effect of head out-degree and tail in-degree on MRR, for the ConvE KGE model.

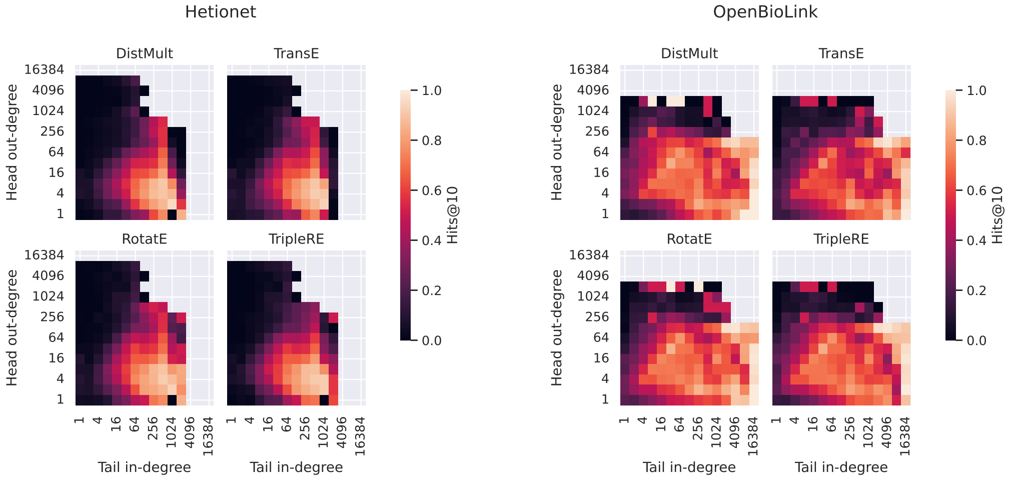

Figure S9: Effect of the head and tail degrees of same relation type on Hits@10, for Hetionet and OpenBioLink.

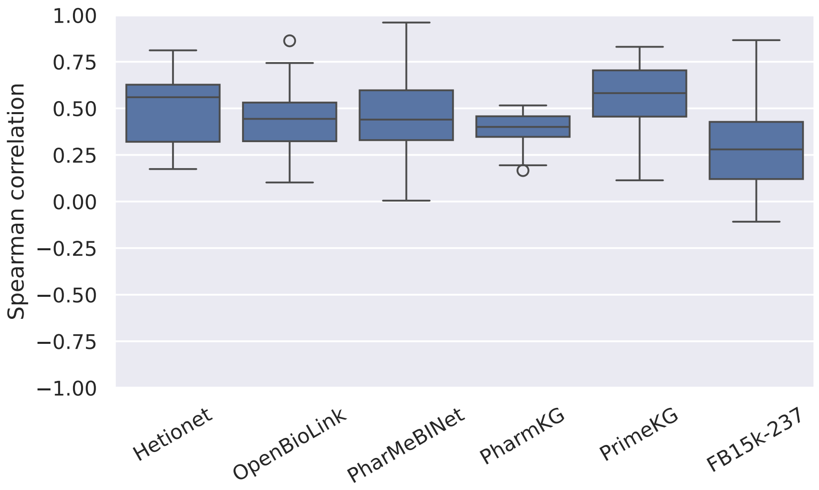

Figure S10: Distribution of Spearman-rank correlation between in-degree of same relation type and how frequently the entity is *incorrectly* selected among the top-100 tail predictions, grouping test queries by relation type. A positive correlation means that KGE models are biased towards predicting entities with a larger number of incoming edges of the relation type considered in the query.

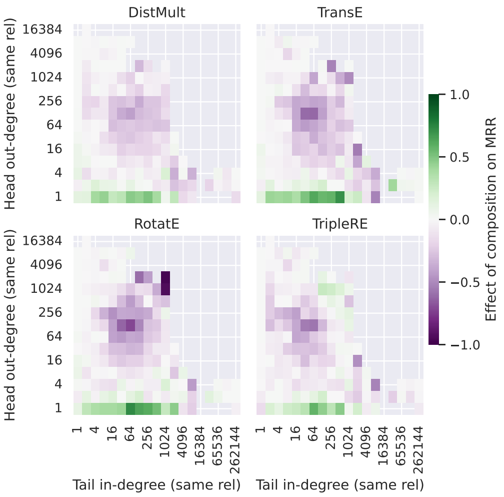

(a) PharMeBINet

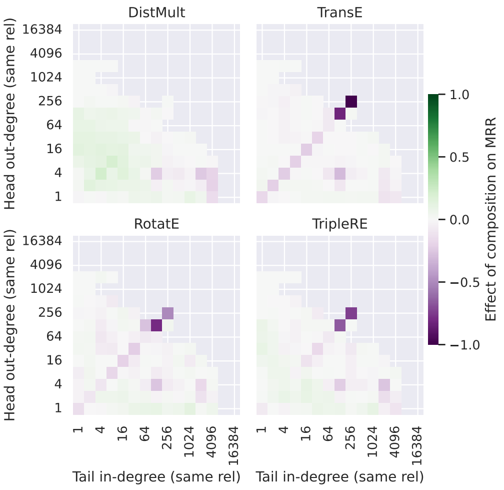

(b) PharmKG

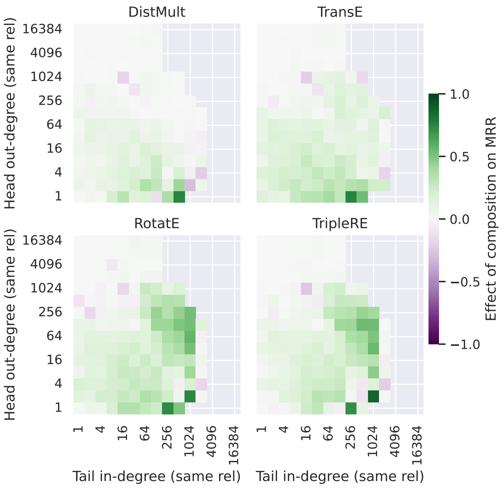

(c) PrimeKG

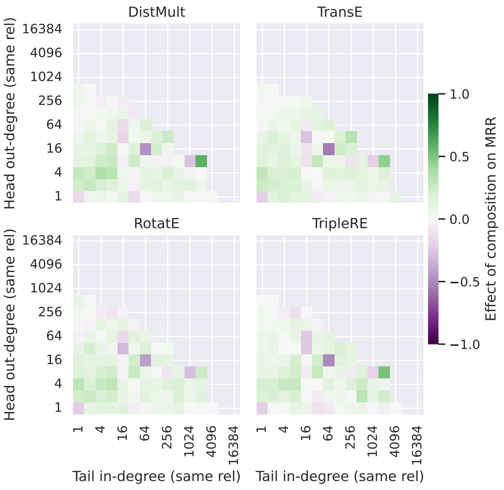

(d) FB15k-237

Figure S11: The effect of having compositions on MRR, triples grouped by their head and tail degrees.

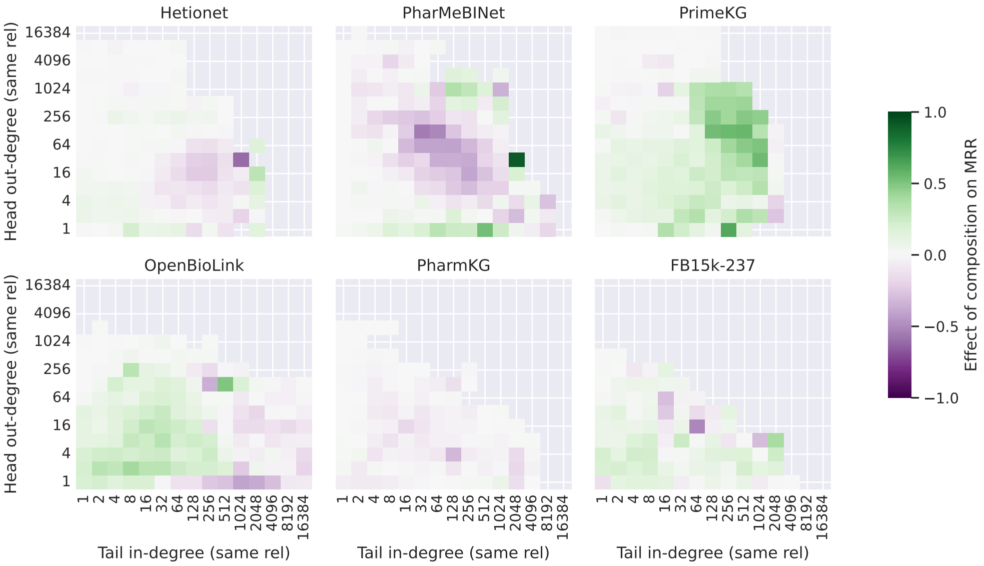

Figure S12: The effect of having compositions on MRR, for the ConvE KGE model.

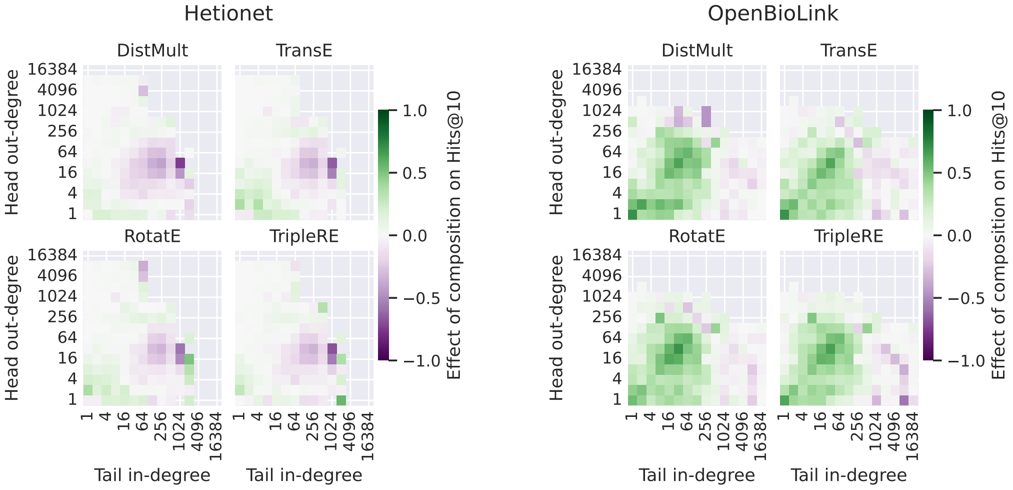

Figure S13: The effect of having compositions on Hits@10, for Hetionet and OpenBioLink.

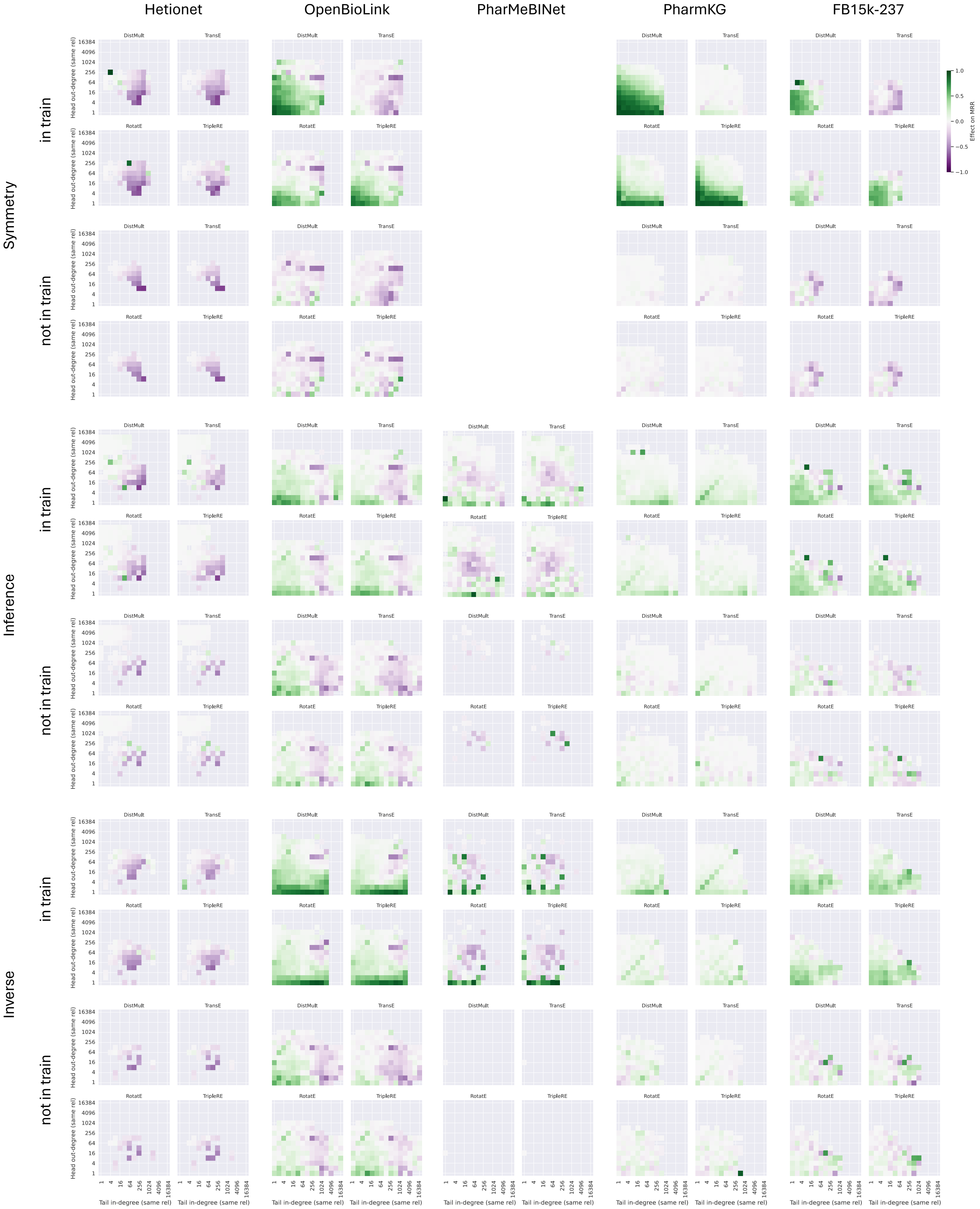

Figure S14: The effect on MRR of being symmetric and having inference/inverse, distinguishing based on whether the counterpart edge is present or absent in in the training data. Note that symmetric triples are too rare in PharMeBINet for a meaningful analysis. Triples grouped by their head and tail degrees.

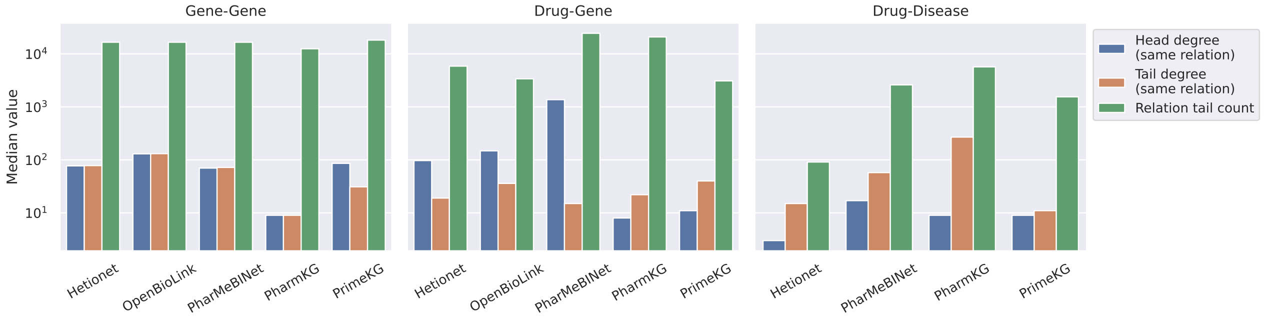

(a) Median head out-degree and tail in-degree of same relation type and number of unique relation tail entities.

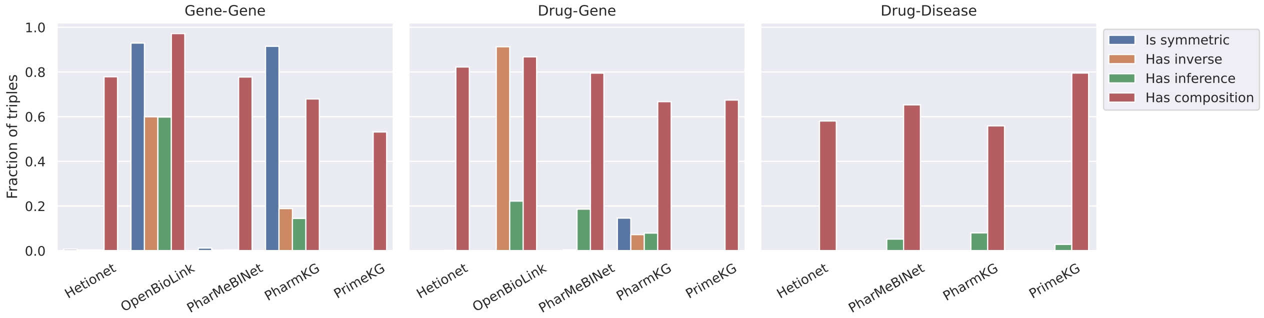

(b) Frequency of edge patterns.

Figure S15: Statistics of topological properties for different interaction types.

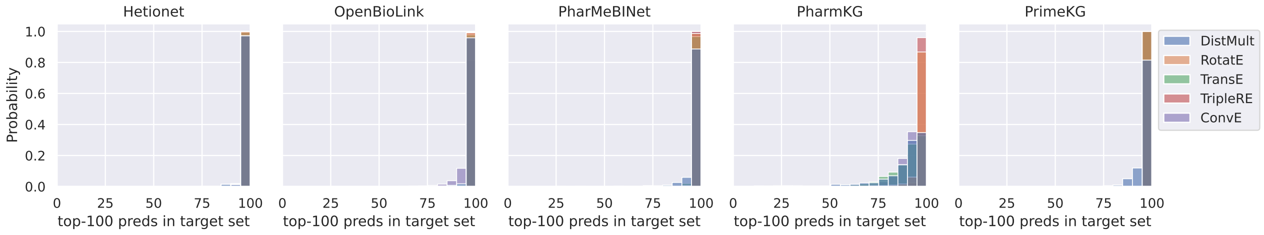

(a) Gene-Gene

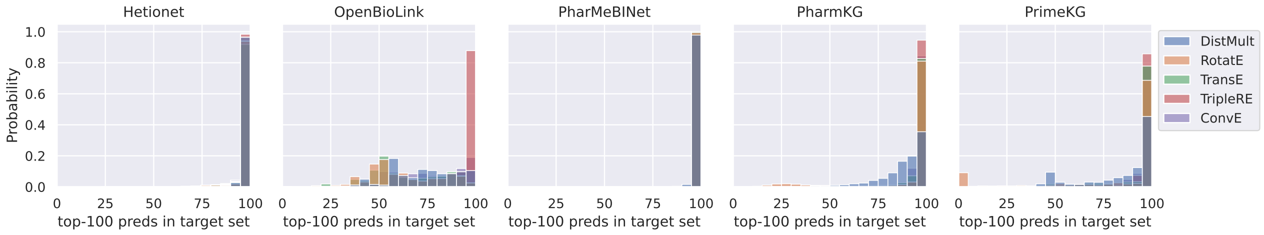

(b) Drug-Gene

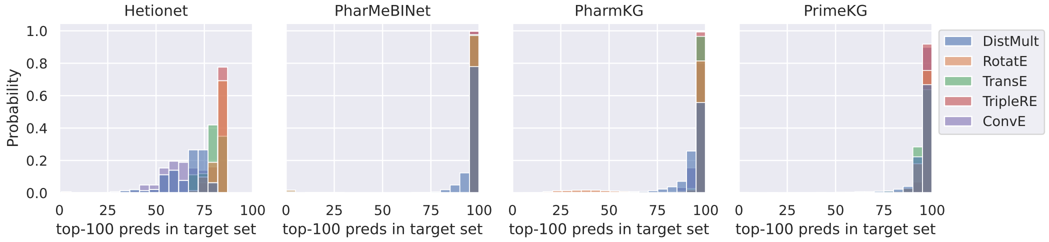

(c) Drug-Disease

Figure S16: Demixing for different interaction types. For each test query, we compute how many of the top-100 predictions made by the model are contained in the set of entities used as tails by triples of the considered relations.

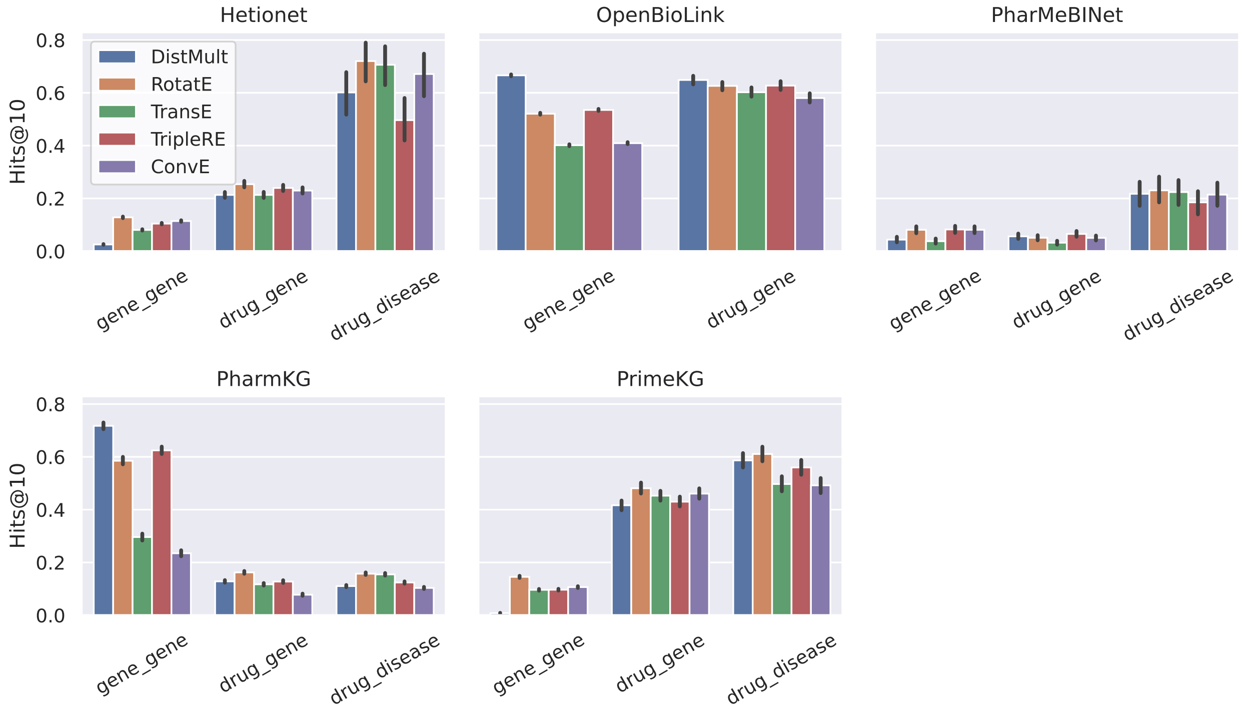

Figure S17: Hits@10 of the interaction types considered in sec. *Predicting Specific Relation Types.*

Table S4: Statistics of the relation types compared between Hetionet and PharMeBINet in sec. *Case Study: Effect of Additional Training Data*. *Matching rate* denotes the fraction of triples of the relation type that are matched to triples in the other dataset. For the rows *Unique heads/tails/out-relations/in-relations* and *Head/Tail out-/in-degree (same relation)*, the median value is reported. For the rows *Has inverse/inference/composition*, we report the fraction of total relation triples with the given property.

**Relation**	**Disease-localizes-Anatomy**		**Compound-binds-Gene**		**Gene-covaries-Gene**		**Anatomy-expresses-Gene**	
**Dataset**	**Hetionet**	**PhMBNet**	**Hetionet**	**PhMBNet**	**Hetionet**	**PhMBNet**	**Hetionet**	**PhMBNet**
Relation triples	3602	3602	11571	11622	61690	61615	526407	526180
Matching rate	1.0	1.0	0.998	0.993	0.999	1.0	0.999	1.0
Test triples	360	360	1141	1141	6161	6161	52618	52618
Unique heads	133	133	1389	1426	9043	9034	241	241
Unique tails	398	398	1689	1701	9542	9518	18094	18074
Head out-degree	212	227	132	2085	74	128	11952	11945
Head out-degree s.r.	34	34	14	14	20	20	7937	7935
Unique out-relations	4	6	4	20	4	8	3	3
Tail in-degree	11	11	102	162	83	114	77	112
Tail in-degree s.r.	11	11	36	36	17	17	44	44
Unique in-relations	1	1	7	13	6	11	6	11
Has inverse	0.0	0.0	0.0	0.03	0.001	0.001	0.0	0.0
Has inference	0.0	0.0	0.006	0.08	0.002	0.002	0.263	0.263
Has composition	0.591	0.594	0.571	0.957	0.501	0.507	0.907	0.907

**Relation**	**Compound-causes-Side Effect**		**Gene-regulates-Gene**		**Gene-interacts-Gene**		**Compound-downregulates-Gene**	
**Dataset**	**Hetionet**	**PhMBNet**	**Hetionet**	**PhMBNet**	**Hetionet**	**PhMBNet**	**Hetionet**	**PhMBNet**
Relation triples	138944	154511	265672	265667	147164	147133	21102	231156
Matching rate	0.909	0.817	0.999	1.0	0.999	1.0	0.997	0.098
Test triples	12630	12630	26566	26566	14713	14713	2105	2105
Unique heads	1071	1358	4634	4634	9526	9525	734	2631
Unique tails	5701	6023	7048	7047	14084	14073	2880	21912
Head out-degree	245	2186	203	254	214	267	515	4144
Head out-degree s.r.	201	182	104	104	54	54	225	1413
Unique out-relations	5	19	6	10	5	10	5	20
Tail in-degree	164	254	309	370	106	145	252	116
Tail in-degree s.r.	164	193	208	208	27	27	20	15
Unique in-relations	1	3	8	12	7	11	8	11
Has inverse	0.0	0.0	0.003	0.003	0.006	0.006	0.0	0.002
Has inference	0.0	0.142	0.006	0.003	0.006	0.006	0.001	0.164
Has composition	0.366	0.893	0.881	0.886	0.703	0.713	0.913	0.800

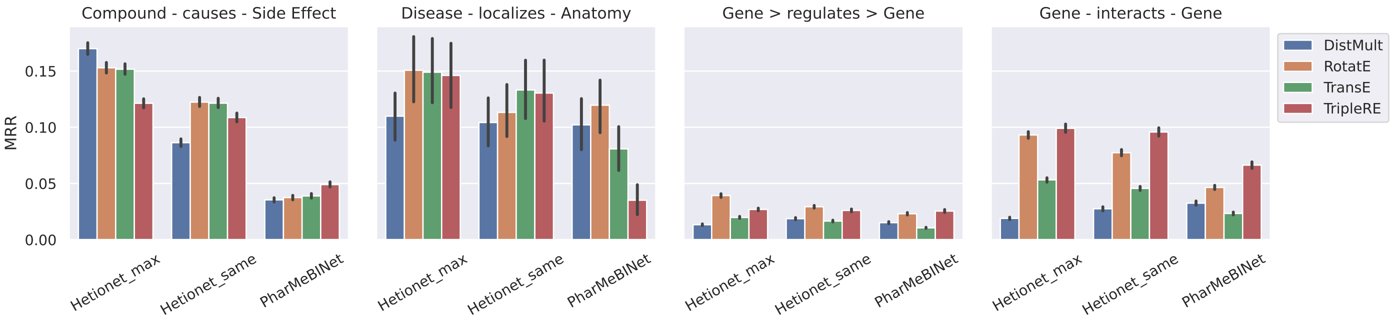

Figure S18: MRR comparison for additional relation types, when testing on a set of common edges between Hetionet and PharMeBINet.

References

Cattaneo A., Justus D., Mellor H., et al. (2022). "BESS: Balanced Entity Sampling and Sharing for Large-Scale Knowledge Graph Completion". In: *arXiv preprint arXiv:2211.12281*.

Kingma D. and Ba J. (2015). "Adam: A Method for Stochastic Optimization". In: *International Conference on Learning Representations (ICLR)*.

Sun Z., Deng Z.-H., Nie J.-Y., et al. (2019). "RotatE: Knowledge Graph Embedding by Relational Rotation in Complex Space". In: *International Conference on Learning Representations*.
